# A slow–fast genotypic difference in metabolic rate shapes thermal responses in honeybees

**DOI:** 10.1098/rsbl.2026.0420

**Published:** 2026-11-11

**Authors:** Elizabeth Rylance, Dhruba Naug

**Affiliations:** Department of Biology, Colorado State University, Fort Collins, USA

**Keywords:** genotypic variation, slow–fast phenotypes, malate dehydrogenase, hermal performance, behaviour, honeybees

## Abstract

Animals face mounting challenges to cope with rapidly changing thermal environments. In honeybees, allelic variation in the cytosolic malate dehydrogenase gene (MDH-1) has been linked to a role in thermal adaptation. The variation is also associated with a difference in metabolic rate that consists of bees with the ‘slow’ allele showing lower metabolic rate than bees with the ‘fast’ allele. To test how this genetic variation influences the thermal performance of traits that are tied to foraging performance, we measured metabolic rate, activity level and learning ability in three MDH-1 genotypes across four temperatures. Metabolic rate showed a significant genotype-by-temperature interaction, activity levels differed by genotype and temperature and learning performance was influenced by genotype but not temperature. These results demonstrate that genotypic variation related to metabolic rate and associated slow–fast variation could be related to differences in thermal performance. We discuss these findings in the context of how functional genetic diversity has implications for collective behaviour and how it can inform breeding strategies for honeybees in the face of rising temperatures.

## 1. Introduction

As global temperatures rise, animals must adjust their physiology and behaviour to respond to increasingly variable thermal environments. These responses may be shaped by phenotypic plasticity, enabling the modification of traits within the same genotype or by evolutionary adaptation involving changes in the frequency of different genotypes over longer time scales [1–4]. Life history theory provides a useful framework for understanding animal responses to environmental changes, as species exhibit a spectrum of strategies that influence their survival, growth and reproduction [5]. More recently, life history strategies have been categorized along a slow–fast continuum, with metabolic rate generally considered to be the fundamental driver of such variation [6]. In this framework, individuals with low metabolic rate reflect a ‘slow’ phenotype and are predicted to have slower growth, delayed reproduction and longer lifespans, while individuals with high metabolic rate represent a ‘fast’ phenotype exhibiting rapid growth, early reproduction and shorter lifespans [7–9].

Given the integral link between temperature and metabolic rate, particularly in ectotherms, recent empirical and theoretical work has suggested that slow–fast phenotypic differences could be related to the thermal physiology of individuals and their performance in different thermal regimes [10–12]. According to this framework, thermal physiology imposes constraints on metabolic rate and thereby sets optimum performance temperatures for behaviours that are linked to maximizing energetic intake, such as locomotion and exploration. This link between temperature and behaviour therefore predicts fast behavioural and life-history traits being associated with warmer environments and slow traits with cooler environments [13,14]. These phenotypic differences may in part arise from underlying gene-environment interactions in terms of the genetic variation in metabolic enzymes and temperature [15–19]. One such key enzyme is the cytosolic malate dehydrogenase (MDH-1), which is a part of the malate-aspartate shuttle that supplies NADH from the cytoplasm to the mitochondria. Populations of plants and invertebrates in warmer environments have MDH-1 allozymes that show higher thermal stability and greater enzymatic performance at elevated temperatures [20–23], but the implications of these biochemical differences on behavioural performance are not as well studied. The repeated emergence of these thermal clines in MDH-1 across taxonomic groups points to its significant role in thermal adaptation.

Honeybees are essential pollinators whose ability to forage in a wide range of thermal environments is key to their success in contributing to the global food supply, particularly under climate change [24,25]. In four continents, Europe, Australia, North and South America, feral populations of the European honeybee *Apis mellifera* show a genotypic variation in MDH-1 that is associated with a latitudinal gradient and its related thermal cline [26–29]. The different variants of the enzyme differ in kinetic properties and thermal stability, leading to differences in their catalytic efficiency at different temperatures [30]. This genotypic variation in MDH-1 is also associated with differences in metabolic rate, with the electrophoretically slow (S) allele linked to a lower metabolic rate and the fast (F) allele linked to a higher metabolic rate [31,32]. MDH-1 allelic variation and the associated difference in metabolic rate are also linked with distinct differences in various behavioural and life history traits that align with a slow–fast phenotypic axis as well as in their thermoregulatory performance [33,34]. However, the difference in metabolic rate between the MDH-1 genotypes did not show a significant interaction with temperature in heterozygotes, suggesting that testing the homozygotes, which have a greater difference in metabolic rate, might reveal such an interaction [35]. Together, these patterns suggest that MDH-1 variation is a key factor that underlies the interaction between thermal physiology and behavioural traits in honeybees and understanding the details of this interaction can provide critical insights into their adaptive significance under rising global temperatures and increasing thermal variability.

To investigate how MDH-1 genetic variation interacts with temperature to influence behavioural responses in honeybees, we tested the performance of three genotypes (SS and FF homozygotes and SF heterozygotes) across four temperatures (24°C, 28°C, 32°C and 36°C), which were selected to span an ecologically relevant range of ambient conditions. We specifically did not measure performance at even higher temperatures that lie beyond the thermal optima of honeybees. We measured three performance traits, metabolic rate, activity level and learning ability, which are linked to differences between slow–fast phenotypes [6,35] and are central to the foraging success of honeybees [32,33,36]. Together, these traits capture thermal performance across the physiological, locomotor and cognitive demands of foraging, allowing us to assess whether genotypic variation in metabolic rate has downstream consequences at the functional level. We hypothesized an interaction between genotype and temperature, predicting that homozygous Fast and Slow individuals would respectively perform better at higher and lower temperatures, and heterozygous individuals would exhibit an intermediate phenotype. We expected this relationship to be strongest for metabolic rate owing to the direct impact of MDH-1 variation on enzyme activity, and potential downstream effects on activity level and learning ability based on previous studies linking these traits within a slow–fast behavioural framework in honeybees [33,36].

## 2. Methods

### (a) Establishing SS, SF and FF colonies

Using the Pol-line stock of *A. mellifera* in the USDA-ARS apiaries in Baton Rouge, Louisiana, USA, protein electrophoresis was used to identify MDH-1 genotypes [30,37]. Brother drones of known genotypes were used to artificially inseminate unrelated virgin sister queens of known genotypes to create the following four types of crosses: (i) S × SS, (ii) F × FF (hereafter called SS and FF for the respective types of brood they produce), (iii) S × FF, and (iv) F × SS (hereafter called SF). This breeding design ensures that the resulting workers share a common genetic background and are expected to differ only at the MDH-1 and any other tightly linked loci. Colonies of each genotype (5 FF, 6 SS and 4 SF) were then set up using worker bees from standard packages (starter colonies) of *A. mellifera* and a queen of a specific genotype. Brood frames at late pupal stage were extracted from these source colonies and placed overnight in an incubator at 32°C and newly hatched worker bees were marked on the abdomen with a dot of paint to track its genotype, source colony and age, and introduced into a single foster colony headed by a wild-type queen to provide a common garden environment.

### (b) Temperature treatment

Marked foragers were collected at 14–18 days of age, individually harnessed inside plastic straws and then placed in a holding room at 30°C (the midpoint of the tested temperature range) for 2 hours. Bees were then tested for the three parameters of interest between 10.00 and 14.00 h at one of the four test temperatures (24°C, 28°C, 32°C or 36°C) using a climate-controlled chamber (AC Infinity), within which temperature was maintained within 0.5°C and humidity was kept between 40–50%. A total of 459 bees were tested in batches of seven bees of a specific genotype tested on a given day for all the three parameters in a fixed order at one of the four temperatures. Each bee was therefore tested at only one of the four temperatures and constitutes a statistically independent replicate.

### (c) Metabolic rate

First, metabolic rate was measured under red light using flow-through CO₂ respirometry with a FoxBox system with an attached multiplexer (Sable Systems). Each harnessed bee was placed inside a 50 ml plastic respirometry chamber and allowed 20 minutes for acclimatization. Ambient air, scrubbed of H₂O and CO₂, was supplied at a constant flow rate of 250 ml min^–1^. The CO₂ concentration in the outgoing airstream was recorded in parallel from all the chambers for 150 s at a time in a pseudorandom sequence that repeated three times for a total of 450 s per bee. A baseline was measured for each run by recording from an empty chamber for 60 s, the mean of which was used to calculate corrected CO_2_ output values for each bee. Resting metabolic rate for each bee was then calculated based on the continuous 2 minutes with the lowest variance in CO₂ production. These values were converted to a power output (mW) using an energy equivalence of 21.4 J ml^–1^ CO₂ and then corrected for body mass, measured at the end of all the assays, to give a mass-specific metabolic rate [38]. We measured resting rather than flight metabolic rate for obtaining a larger sample size as previous data show resting and flight metabolic rates of a bee to be highly correlated [33].

### (d) Activity level

Following the measurement of metabolic rate, bees were released from their restraints into activity monitoring tubes (16 mm diameter) and their locomotor activity was recorded for 60 minutes using an automated infrared-based system (TriKinetics System, LAM16). During this assay, bees had unrestricted access to 40% sucrose provided in cotton plugs at both ends of the tube. To determine the peak activity level for each bee, we used a rolling window to identify the 15 consecutive minutes with the highest total counts for a bee. We used locomotor activity as a convenient proxy for flight activity as the two are generally correlated [39,40].

### (e) Learning performance

Following the activity assay, bees were assayed for their learning performance using an aversive conditioning assay with a shock avoidance apparatus [41], in which bees learn to avoid a colour cue (the conditioned stimulus, CS) associated with a mild electric shock (the unconditioned stimulus, US) as punishment. Bees were moved from the activity tubes into the apparatus and allowed to adjust without the CS or US for 10 minutes. Bees were then subjected to two 5-minute training sessions, during which the US was paired with the CS, separated by a 10-minute inter-training interval (ITI). Following the second training period, bees were tested after 10 minutes for successful avoidance learning in the absence of the US. Bees were placed in the centre of the apparatus and by videotaping these test sessions, their position was scored every 15 s for 1 minute with respect to whether they exhibited the correct conditioned response (avoidance of the colour cue associated with the ‘shock side’ of the grid). A correct response was scored as 1, and an incorrect response was scored as 0. Learning performance for each bee was calculated as the proportion of correct conditioned responses during the test period.

### (f) Statistical analysis

All analyses were performed in R (packages: lme4, stats, emmeans). To assess potential effects of source colony, mixed-effects models were constructed with colony nested within genotype as a random intercept and these were compared to original models without random effects using AIC model comparison. Since source colony did not improve model fit, it was excluded from further analysis (see supplementary material, table S1 for details). For metabolic rate and activity, extreme outliers in the 99th percentile were excluded and for the learning assay, bees that did not walk on the shock grid, thereby avoiding the US, were excluded from the analysis, resulting in slightly different sample sizes for the three parameters. Separate models were constructed for metabolic rate, activity level and learning performance, with genotype (FF, SF and SS) and temperature (24°C, 28°C, 32°C and 36°C) as predictors. The metabolic rate data exhibited heteroscedasticity, with increasing variance at higher temperatures, and a generalized linear model with a gamma distribution and log link provided the best fit. The activity level data were approximated by a normal distribution and were analysed using a linear model. Learning performance was analysed using a generalized linear model with binomial distribution and logit link. Model terms were evaluated using likelihood ratio chi-squared tests. Orthogonal polynomial contrasts were applied to assess linear and quadratic components of the temperature effect. Estimated marginal means and Tukey-adjusted pairwise comparisons were used for post hoc analysis of genotype and temperature effects.

## 3. Results

### (a) Metabolic rate

Metabolic rate was significantly affected by both temperature (χ² = 59.13, d.f. = 3, *p* < 0.001) and genotype (χ² = 21.32, d.f. = 2, *p* < 0.001), as well as their interaction (χ² = 15.15, d.f. = 6, *p* < 0.05). Genotypic differences were observed at 24°, 28° and 36°C (figure 1A; supplementary material, table S2A), with FF bees exhibiting higher metabolic rates than SS bees and SF bees being intermediate. Notably, metabolic rates of SF bees shifted from being more SS-like at low temperatures to being more FF-like at high temperatures. The linear component of the temperature effect was significant for all three genotypes (FF: *t* = 3.18, *p* < 0.01; SF: *t* = 2.06, *p* < 0.05; SS: *t* = 2.91, *p* < 0.01), consistent with the increasing metabolic rates observed across the temperature range (figure 1A). However, SS bees also showed a significant quadratic component of the temperature effect (*t* = 2.46, *p* < 0.05), which suggests that the metabolic rate of SS bees may have reached a peak within the tested temperature range—a pattern not observed for SF or FF bees. When averaged over all temperatures, FF and SF bees had higher metabolic rates than SS bees, but SF bees did not differ significantly from FF bees (figure 1B; supplementary material, table S3). When averaged over all genotypes, bees tested at 28°, 32° and 36°C had significantly higher metabolic rates than bees tested at 24°C (figure 1B; supplementary material, table S3). Additionally, bees at 32° and 36°C had significantly higher metabolic rates than those at 28°C. However, metabolic rates at 32° and 36°C did not differ significantly from each other.

**Figure 1.**
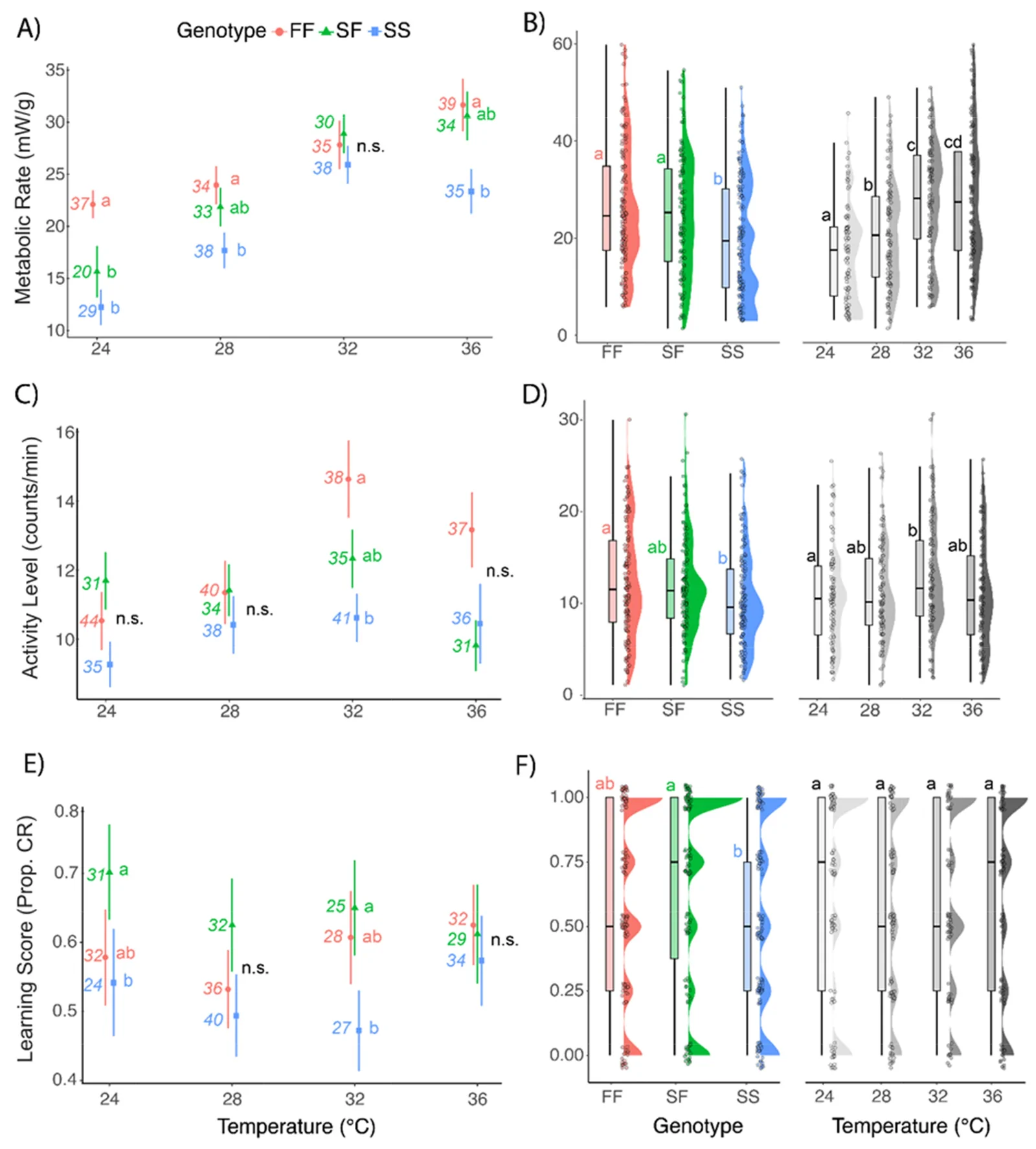
Effects of MDH-1 genotype and temperature on (A,B) metabolic rate, (C,D) activity level and (E,F) learning score. Figures on the left column (A,C,E) show the genotype-temperature relationship for each response variable with data presented as means with standard error bars and sample sizes in respective colours; significant differences among genotypes within a temperature are denoted by different letters in respective colours (*p* < 0.05) using Tukey-adjusted pairwise comparisons of estimated marginal means. Figures on the right column (B,D,F) show the distribution of each response variable by genotype and temperature with data presented as half-violin (density) plots and boxplots displaying the interquartile range (IQR) and whiskers extending to 1.5 × IQR; significant differences, assessed separately for genotype and temperature, are indicated by different letters in respective colours (*p <* 0.05) from Tukey-adjusted pairwise comparisons of estimated marginal means. Together, the six panels display the complete dataset from a fully crossed design spanning three genotypes, four temperatures and three performance traits.

### (b) Activity level

Temperature (F_3,414_ = 2.93, *p <* 0.05) and genotype (F_2,414_ = 4.16, *p <* 0.05) were both significant predictors of activity, although their interaction was not significant (F_6,414_ = 1.41, *p =* 0.21). Genotypic differences were observed only at 32°C (figure 1C; supplementary material, table S2B), with FF bees having the highest activity and SS bees having the lowest. Orthogonal polynomial contrasts revealed a significant linear component of the temperature effect for the FF (*t =* 2.72, *p <* 0.01) and SF genotypes (*t =* 2.69, *p <* 0.01), indicating that FF activity increased and SF activity decreased linearly with temperature. SS activity did not respond significantly to changes in temperature (*t =* 1.49, *p =* 0.13). The quadratic component was not significant for any genotype (FF: *t =* 1.32, *p =* 0.19, SF: *t =* 0.01, *p =* 0.98, SS: *t =* 0.38, *p =* 0.69). When averaged over temperatures, FF bees had higher overall activity levels than SS bees, while SF bees did not differ significantly from either FF or SS bees (figure 1D; supplementary material, table S3). When averaged over genotypes, activity at 32°C was significantly higher than those at 24°C, but no other pairwise temperature comparisons were significant.

### (c) Learning performance

Genotype contributed significantly to learning performance (χ² = 16.73, d.f. = 2, *p <* 0.0015), but temperature did not (χ² = 3.16, d.f. = 3, *p =* 0.37), nor did a genotype-by-temperature interaction (χ² = 4.88, d.f. = 6, *p =* 0.56). Genotypic differences were observed at 24°, 28° and 32°C; pairwise contrasts indicated that SF bees outperformed SS bees overall, while no significant differences in learning were observed between FF and SF or between FF and SS genotypes (figure 1E; supplementary material, table S2C). There were no significant linear (FF: *z =* 1.10, *p =* 0.27; SF: *z =* –1.69, *p =* 0.08; SS: *z =* –0.51, *p =* 0.61) or quadratic trends (FF: *z =* 0.71, *p =* 0.47; SF: *z =* –0.21, *p =* 0.83; SS: *z =* 0.65, *p =* 0.51) in learning performance across temperatures. When averaged across temperatures, SF bees exhibited higher learning performance than SS bees but did not differ significantly from FF bees. Learning performance also did not differ between FF and SS bees when averaged across temperatures (figure 1F; supplementary material, table S3). There were no significant pairwise differences between temperatures when averaged across genotypes.

## 4. Discussion

Honeybees are vital pollinators that forage in highly variable thermal environments, and this study provides experimental evidence that genotypic variation at the MDH-1 locus contributes to temperature-dependent performance differences, with important implications for honeybee foraging. It is particularly striking that a single polymorphism gave rise to such pronounced phenotypic differences across multiple traits. We found that genotypes associated with distinct slow–fast phenotypes responded differently to temperature in terms of their metabolic rates, activity levels and learning performance. However, the strength and pattern of temperature effects varied by trait, with metabolic rate and activity showing stronger responses to temperature than learning. As predicted, metabolic rate showed a clear genotype-by-temperature interaction, with homozygous Fast bees (FF) exhibiting consistently higher rates than homozygous Slow (SS) bees and heterozygous bees showing intermediate rates. The quadratic trend for metabolic rate observed in homozygous Slow bees but not in homozygous Fast or heterozygous bees implies a potential metabolic performance peak for Slow bees within the tested temperature range. This pattern is consistent with research showing that slow phenotypes have lower thermal optima than fast phenotypes [10,12]. Activity level showed no genotype temperature interaction, but each of them independently influenced activity. Consistent with the effect on metabolic rate, Fast bees had significantly higher activity levels than Slow bees, and their activity increased linearly with temperature. Learning performance was influenced by genotype but not temperature and we found no evidence of an interaction between the two. We expected learning performance to be higher in Fast bees based on previous research showing faster learning linked to higher metabolic rate [42], but it is possible that learning being an energetically expensive process might lead to its higher impairment in the FF genotypes when their metabolic rate is exceedingly high at higher temperatures.

The heterozygous (SF) bees proved to be the most intriguing case. They displayed a temperature-dependent shift in metabolic rate, resembling the Slow phenotype at cooler temperatures and the Fast phenotype at warmer temperatures. This pattern could reflect greater thermal sensitivity in the heterozygotes, indicated by a steeper reaction norm, and suggest a flexible strategy for context-dependent trait expression. Consistent with this, the heterozygotes showed a decrease in activity levels at higher temperatures, potentially suggesting a more flexible behavioural strategy to limit activity under thermal stress. Biochemical and structural analyses have shown that the F and S MDH-1 allozymes differ in both catalytic efficiency and thermal stability in honeybees and that SF heterodimers maintain high activity across a broad temperature range [30]. The heterozygotes also showed a higher learning performance across the temperature range. This broad thermal performance may potentially confer a heterozygote advantage, allowing for higher plasticity in variable thermal environments, considered a critical part of the current thermal landscape [43].

Our results suggest that genotypic variation in MDH-1 could contribute to colony-level plasticity through its effects on individual thermal responses. Genetically based differences in response thresholds stabilize colony-level functions such as thermoregulation, allowing for more effective responses to environmental perturbations [44] and it is well known that such individual-level differences in physiology and behaviour can show complex scaling effects [45]. Genetic diversity has been shown to positively influence colony fitness in honeybees [46–49], but the potential for genetic diversity to produce negative or neutral effects on collective performance is less frequently considered [50–53]. Our results imply that genetic variation in traits associated with the slow–fast phenotypic continuum could shape colony-level responses to thermal change in complex ways. Such variation may facilitate adaptive plasticity, but could also introduce challenges if mismatches in response disrupt collective performance.

Despite the significant influence of genetic variation on thermal performance observed in our study, the potential significance of such variation has not been sufficiently appreciated in current honeybee management practices. Honeybee populations in North America and Europe are highly managed, and current breeding practices have led to most colonies typically being sourced from a narrow genetic pool [54]. As a result, these populations may not necessarily be optimized in terms of their metabolic and thermal phenotypes for their local climates. By experimentally demonstrating that MDH-1 genotypes differ in the thermal sensitivity of traits that are relevant to foraging and colony performance, our findings highlight the importance of understanding this genetic variation in more detail, not only for addressing fundamental questions in behavioural evolution but also for potentially informing future honeybee breeding practices in the face of climate change.

## Supplementary Material



## Data Availability

Data related to the study is uploaded as supplementary material. Supplementary material is available online [55].
